# CoMn_2_O_4_ Catalyst Prepared Using the Sol-Gel Method for the Activation of Peroxymonosulfate and Degradation of UV Filter 2-Phenylbenzimidazole-5-sulfonic Acid (PBSA)

**DOI:** 10.3390/nano9050774

**Published:** 2019-05-20

**Authors:** Chihao Lin, Dejian Shi, Zhentao Wu, Lingfeng Zhang, Zhicai Zhai, Yingsen Fang, Ping Sun, Ruirui Han, Jiaqiang Wu, Hui Liu

**Affiliations:** 1 College of Biological, Chemical Sciences and Engineering, Jiaxing University, Jiaxing 314001, China; lin15967935943@163.com (C.L.); sdj6299@163.com (D.S.); w762601270@163.com (Z.W.); m18035113969@163.com (L.Z.); njuzzc@sina.com (Z.Z.); 2 Nanhu College, Jiaxing University, Jiaxing 314001, China; hanrui826@163.com (R.H.); tntcs1983@163.com (J.W.)

**Keywords:** CoMn_2_O_4_, catalytic degradation, peroxymonosulfate, UV filters, 2-phenylbenzimidazole-5-sulfonic acid (PBSA)

## Abstract

In this study, a bimetallic oxide catalyst of cobalt-manganese (CoMn_2_O_4_) was synthesized using the sol-gel method, and it was then characterized using a variety of techniques such as scanning electron microscopy (SEM), transmission electron microscopy (TEM), X-ray diffraction (XRD) spectroscopy, X-ray photoelectron spectroscopy (XPS), and nitrogen adsorption–desorption isotherms. The obtained novel catalyst, i.e., CoMn_2_O_4_, was then used as an activator of peroxymonosulfate (PMS) for the catalytic degradation of a commonly-used UV filter, 2-phenylbenzimidazole-5-sulfonic acid (PBSA) in water. The effects of various factors (e.g., catalyst dosage, PMS concentration, reaction temperature, and pH) in the process were also evaluated. Chemical scavengers and electron paramagnetic resonance (EPR) tests showed that the ^•^OH and SO_4_^•−^ were the main reactive oxygen species. Furthermore, this study showed that CoMn_2_O_4_ is a promising catalyst for activating PMS to degrade the UV filters.

## 1. Introduction

In recent years, UV filters have been increasingly used in personal care products (PCPs), such as sunscreen creams, lipsticks, shampoos, and hair gels. Given their dramatically increasing daily use, UV filters are continuously being discharged into the aquatic environment, and have become a class of emerging contaminants (ECs) and also formed “false persistent” pollution [1,2]. A large number of studies have shown that some organic UV filters can mimic the effects of various hormones in living organisms, thereby interfering with the normal endocrine functions of animals and humans, resulting in potentially serious health threats [3,4,5,6,7,8]. Unfortunately, UV filters are relatively stable in nature and are considered difficult to be biodegraded, making it difficult for municipal wastewater treatment plants to completely remove them during treatment [9,10]. In view of the yearly increases in production and emission of these ECs, the environmental pollution caused by UV filters have become a research hotspot in the environmental field worldwide. Therefore, it is necessary to apply some cost-effective techniques such as advanced oxidation processes (AOPs), to ensure the efficient elimination of such ECs from the water environment [11].

Over the past few decades, traditional AOPs have mainly focused on hydroxyl radicals (^●^OH) as the main reactive oxidative species (ROS) to oxidize organic pollutants [12]. As is well known, hydrogen peroxide (H_2_O_2_) is commonly used as an oxidant to decompose organic pollutants during the production of ^●^OH under certain conditions [13,14,15]. However, as a liquid, H_2_O_2_ is inconvenient to transport and easily self-decomposes under normal temperature conditions, which results in a low utilization rate. Meanwhile, an increasing number of studies have shown that activated persulfate (PS), including peroxydisulfate (PDS) and peroxymonosulfate (PMS), can produce sulfate radicals (SO_4_^●−^), which have high oxidizing power for the selective degradation of organic pollutants [16,17,18]. Comparatively, as solid chemicals, PS are considered convenient for transportation and storage, and these chemicals are relatively stable at room temperature. Therefore, SO_4_^●−^-based AOPs (SR-AOPs) have been rapidly applied in water pollution control as alternatives to the ^●^OH-based AOPs [19,20,21,22,23,24].

The key to the application of the SR-AOPs is to increase the yield of SO_4_^●−^. Usually, the methods of activating PS have included heat [25], UV [26], and transition metal ions [27]. However, some of these methods can be expensive, whilst others would cause secondary pollution. Heterogeneous catalysts for PS activation such as Fe_3_O_4_ have become the focus of current research because they do not pose a secondary pollution problem, have a fast activation rate at normal temperature and pressure, and the process does not require additional energy consumption [28,29]. These types of catalysts have the advantages of environmental friendliness, easy magnetic separation, and low cost. However, their catalytic effects are not satisfactory, and drawbacks also exist, such as low catalytic activity, low oxidant utilization rate, and incomplete degradation of the organic intermediates. In order to improve the catalytic performance, different transition metals, such as Cu, Mn, Cr, Co, etc., have been added to the Fe_3_O_4_ [30,31]. Alternatively, humic acid, EDTA, polyhydroquinone, etc., have been coated on the surface of the Fe_3_O_4_ [32,33,34,35,36,37,38]. Manganese, a multi-use metal with many stable oxides, has also been used in bimetallic oxides together with Co where it exhibits higher catalytic ability than Co_3_O_4_, Mn_2_O_3_, and their mixtures for PMS activation to degrade Rhodamine B [39]. However, very few systematic studies have been reported on the heterogeneous catalysts containing Mn, as well as their catalytic activities on PMS. Therefore, these composite materials may be promising candidates for the SR-AOPs [40].

This work was aimed at studying the performance of CoMn_2_O_4_ in activating PMS for the degradation properties of a common UV filter, 2-phenylbenzimidazole-5-sulfonic acid (PBSA) [41,42]. Generally, metal oxides could be prepared using several methods, i.e., the coprecipitation, hydrothermal, and sol-gel methods. Amongst them, the sol-gel method is considered an attractive synthetic method in which the prepared metal oxides have a higher degree of structural and compositional uniformity [43,44]. Therefore, in this study, CoMn_2_O_4_ was prepared using the sol-gel method and then it was characterized using the following techniques: SEM, TEM, XRD, BET, and XPS [45]. Moreover, the effects of various factors on the degradation of PBSA were assessed, and the main reactive oxygen species (ROS) in the PMS/CoMn_2_O_4_ system were confirmed as stimulating the activation mechanisms.

## 2. Materials and Methods

### 2.1. Materials

Industrial graphene (reduced graphene oxide, rGO, >97%) and carboxylated carbon nanotube (CNT–COOH, >97%) were obtained from Timesnano (Chengdu, China). Co_3_O_4_ (99.5%, 30 nm) and Mn_3_O_4_ (97%) were obtained from Macklin Biochemical Co., Ltd. (Shanghai, China). PBSA (97%) was obtained from J&K (Shanghai, China) and PMS was obtained from Aladdin (Shanghai, China). Methanol and formic acid were of HPLC grade obtained from Sigma-Aldrich (Shanghai, China), and other reagents were of an analytical grade and they were obtained from Macklin Biochemical Co., Ltd. (Shanghai, China), including Co(NO_3_)_2_·6H_2_O, Mn(NO_3_)_2_∙4H_2_O, citric acid, glycine, humic acid (HA), absolute ethanol (EtOH), and tert-butanol (TBA).

### 2.2. Preparation of the CoMn_2_O_4_ Catalyst

The CoMn_2_O_4_ catalyst was prepared using the sol-gel method as described in Reference [44]. Typically, Co(NO_3_)_2_∙6H_2_O (0.015 mol) and Mn(NO_3_)_2_∙4H_2_O (0.030 mol) were dissolved in 90 mL of water, and then glycine (0.045 mol) was added. The obtained solution was then stirred and placed in a water bath at 80 °C until it was completely dissolved. Afterwards, citric acid (0.045 mol) was slowly added to the solution, which was stirred until a gel was formed. The obtained wet gel was then placed in an oven and dried at 250 °C for 1 h. Subsequently, the obtained dry gel was ground and placed in a muffle furnace, and then calcined at 500 °C for 2 h at 10 °C/min to prepare the CoMn_2_O_4_ nanopowders.

### 2.3. Characterization Methods

The surface elemental composition of the sample was analyzed by the ESCALAB 250XI X-ray photoelectron spectroscopy (XPS) (Thermo Fisher Scientific, Waltham, MA, USA) using Al K-alpha radiation under conditions optimized for the maximum signal (spot size, 500 µm; lens mode, standard; analyzer mode, CAE; pass energy 30.0 eV; energy step size, 0.050 eV). Wide scans were recorded for the CoMn_2_O_4_, whilst the core level peaks that were recorded in detail were: C 1s and O 1s, Co 2p and Mn 2p.

The morphologies were determined using the Quanta400FEG scanning electron microscope (SEM, FEI, Hillsboro, OR, USA) at 20 kV and the JEM-2100F transmission electron microscope (TEM, JEOL, Tokyo, Japan). The elemental composition was determined using a Horiba EX-250 energy-dispersive X-ray (EDX, Kyoto, Japan) at 20 kV.

The crystal structure of the synthesized sample was confirmed through the X-ray diffraction spectra recorded in the 2θ range of 5–80° (scan rate of 0.06° s^−1^), using a Cu–Kα (λ = 0.154 nm) wavelength D8-advanced X-ray diffractometer (XRD, Bruker, Karlsruhe, Germany) at 40 kV and 30 mA.

The specific surface area and the pore size distribution were determined using the TriStar II 3020 surface area and porosity analyzer (Micromeritics, Atlanta, GA, USA) at the liquid nitrogen temperature (−196 °C).

### 2.4. Catalytic Test Procedure

Thereafter, 100 mL PBSA solution (5 mg/L) was added in a conical flask. A certain amount of PMS was then added to the reaction solution, and the mixture was shaken in a water bath at 25 °C. The catalyst CoMn_2_O_4_ was then added to initiate the reaction. Then, a 0.8 mL solution was sampled with a pipette at defined time intervals, filtered through a 0.45 μm microporous membrane, and injected into a vial of high-performance liquid chromatography (HPLC). The vial was filled with 0.2 mL of methanol as a quencher. The solution concentration was measured using HPLC.

### 2.5. Analytical Methods

The concentration of PBSA in the sample was analyzed by a Shimadzu LC-20A HPLC with a diode array detector (DAD). The specific conditions: an Agilent Zorbax SB-C18 column (4.6 × 250 mm, 5 μm, Santa Clara, CA, USA) was used; the mobile phase was a MeOH and formic acid solution (0.30%); the flow rate was 1.0 mL/min; the injection volume was 20 μL; and the quantitative wavelength was 303 nm.

Total organic carbon (TOC) was determined using a Liqui TOC II analyzer (Elmentar, Frankfurt, Germany). Reactive oxidative species (ROS) generated in the CoMn_2_O_4_/PMS system were tested using a Bruker A320 electron paramagnetic resonance (EPR, Karlsruhe, Germany) spectroscopy with 5,5-dimethy-1-pyrroline (DMPO) as a spin-trapping agent as described in Reference [23]. The parameters were: center field, 3510.0 G; sweep width, 100.0 G; static field, 3410.0 G; microwave frequency, 9.85 GHz; microwave power, 18.94 mW; modulation frequency, 100.0 G; modulation amplitude, 1.0 G; time constant, 10.24 ms; conversion time, 30 ms; and sweep time was 30.72 s.

## 3. Results and Discussion

### 3.1. Characterization of the Catalyst

**Figure 1:**
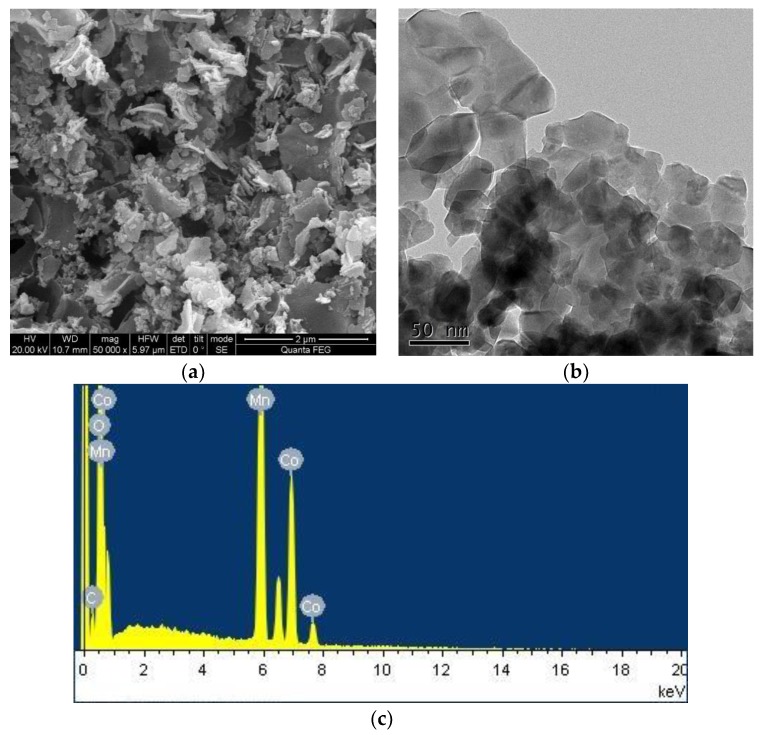
Scanning electron microscopy (SEM) (**a**), transmission electron microscopy (TEM) (**b**) images, and energy-dispersive X-ray (EDX) analysis (**c**) of CoMn_2_O_4_.

The morphology and structure of the CoMn_2_O_4_ were revealed by the SEM and TEM images. As shown in Figure 1a, it can be seen that the sample was in the form of irregular flakes with a uniform distribution, where fine particles were distributed on the edge of the block. There was a certain agglomeration and fluffy accumulation, as well as many pores between the particles. As presented in Figure 1b, it can be seen that the grains of the CoMn_2_O_4_ powder had an irregular polyhedral structure. Furthermore, the energy-dispersive X-ray (EDX) elemental analysis spectrum of the CoMn_2_O_4_ in Figure 1c indicated that the catalyst contained C, O, Co, and Mn elements. The content of C, O, Co, and Mn was calculated with the average of four values on different spots, and their values were 5.97, 35.08, 19.48, and 39.46 wt % (Weight %), respectively, as listed in Table 1.

**Figure 2:**
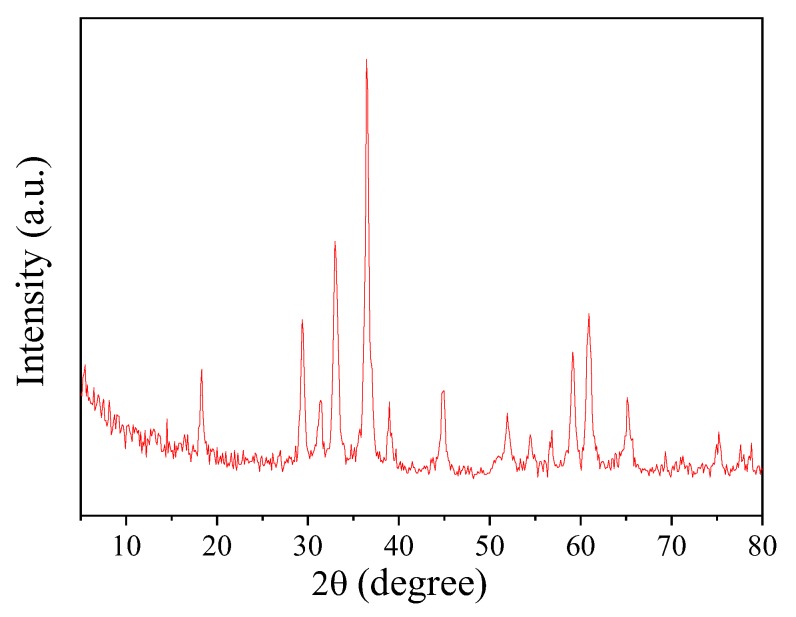
X-ray diffraction (XRD) pattern of CoMn_2_O_4_.

The crystal structure of the CoMn_2_O_4_ nanoparticle was examined using XRD, as shown in Figure 2. The diffraction peaks appeared at 18.2°, 29.3°, 31.2°, 32.8°, 36.3°, 38.7°, 44.7°, 51.8°, 54.3°, 56.5°, 59.0°, 60.6°, 65.1°, and 74.9°, respectively, which was consistent with the CoMn_2_O_4_ crystalline structure (JCPDS 77-0471) as in Reference [30]. Moreover, no other peaks were observed on the XRD pattern of the sample, indicating the high purity of the CoMn_2_O_4_ obtained using the sol-gel method.

**Figure 3:**
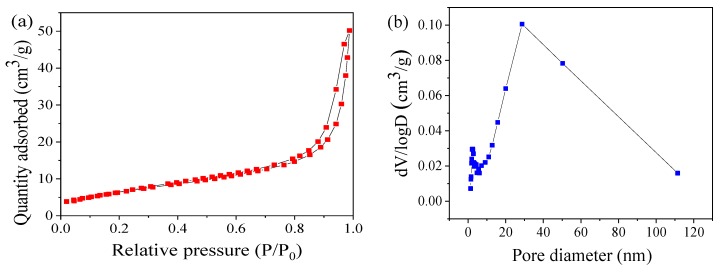

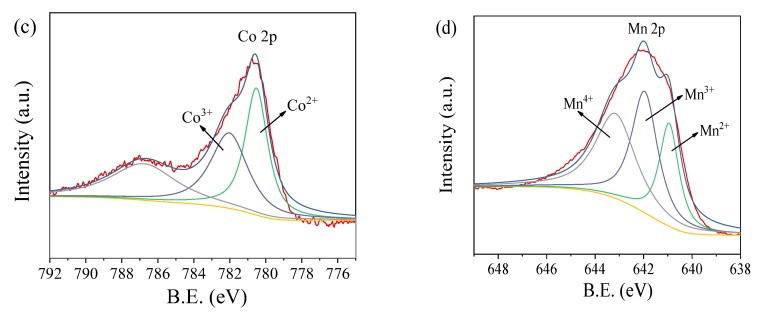
N_2_ adsorption–desorption isotherms (**a**) and pore size distributions (**b**); Co 2p (**c**) and Mn 2p (**d**) spectrum of CoMn_2_O_4_.

As shown in Figure 3a, the nitrogen adsorption–desorption isotherm displays a type IV isotherm with a wide H3 hysteresis area, indicating the existence of mesopores. This can be further revealed through the corresponding pore–size distribution plots, which was calculated using the BJH (Barrett-Joyner-Halenda) method from the desorption branch isotherm as shown in Figure 3b. These pores may be formed by the agglomeration and fluffy accumulation of the CoMn_2_O_4_ particles, as shown in the SEM and TEM images (Figure 1a,b). The specific surface area (SSA) of CoMn_2_O_4_ was approximately 24.23 m^2^ g^−1^, which was calculated using the multipoint BET (Brunauer-Emmett-Teller) method. Figure 3c,d displays XPS surveys of the elemental compositions and chemical states of the CoMn_2_O_4_, respectively. Two components of Co were found in the CoMn_2_O_4_, that is Co^2+^ at 780.5 eV and Co^3+^ at 782.0 eV (Figure 3c). Mn existed in three forms, that is, Mn^2+^, Mn^3+^, and Mn^4+^ at 641.1, 642.1, and 643.4 eV, respectively (Figure 3d). This indicated that the Co and Mn species in the CoMn_2_O_4_ existed as mixed valences. These results were consistent with the features of spinel-type CoMn_2_O_4_. Thus, the above characterizations confirmed the successful preparation of the CoMn_2_O_4_ nanomaterial by the sol-gel method.

### 3.2. Catalytic Oxidation of the PBSA

**Figure 4:**
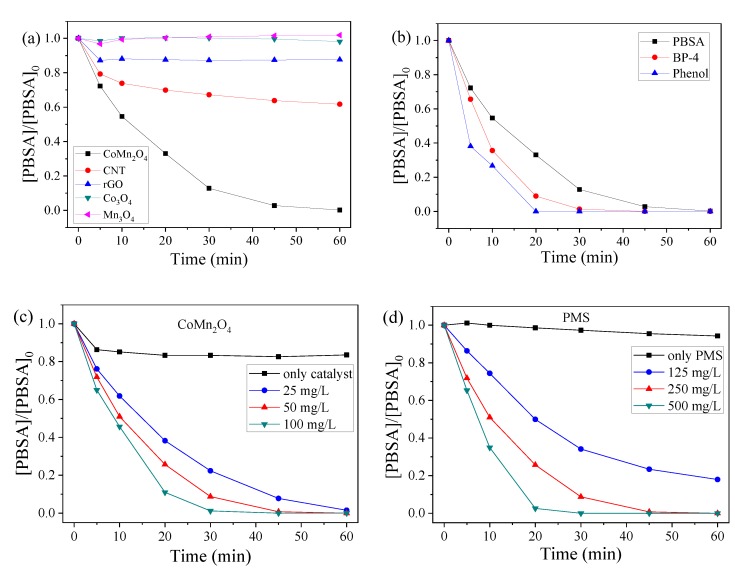

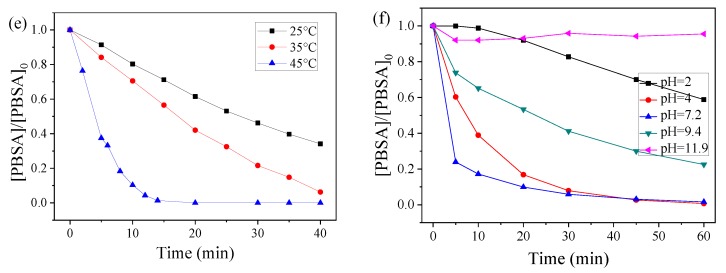
Influences of the different catalysts (**a**), CoMn_2_O_4_ dose (**c**), PMS dose (**d**), reaction temperature (**e**), and pH on the PBSA removal (**f**) and BP-4 and phenol removal (**b**) by the CoMn_2_O_4_/PMS system. Conditions: [PBSA] = 5 mg L^−1^; [BP-4] = [phenol] = 18.23 mM; [catalyst] = 50 mg L^−1^ (10 mg L^−1^, Figure **e**); [PMS] = 250 mg L^−1^; *T* = 25 °C; and without pH adjustment.

To investigate the activation efficiencies of various catalysts/PMS systems, dynamic experiments to remove the PBSA were conducted as shown in Figure 4. From Figure 4a,b, neither the conventional nanocarbons (CNT–COOH and rGO) nor the classical metal catalysts (Co_3_O_4_ and Mn_3_O_4_) were effective in activating PMS to remove the PBSA under the selected conditions. Comparatively, the CoMn_2_O_4_/PMS system not only exhibited considerable removal efficiency of the PBSA but also showed a universality for the degradation of other pollutants (e.g., UV filter benzophenone-4 (BP-4) and phenol).

The dosage of the catalysts is a key index in the process of oxidative degradation. Figure 4c shows the effect of different amounts of CoMn_2_O_4_ on the degradation of the PBSA. In the absence of PMS, the CoMn_2_O_4_ could not remove the PBSA effectively even though the concentration of CoMn_2_O_4_ was increased to 50 mg/L. This indicated the weak adsorption of CoMn_2_O_4_ (at only about 16%). With 250 mg/L PMS, the CoMn_2_O_4_ could efficiently activate the PMS to degrade the PBSA, and the degradation reaction conformed to the pseudo-first-order kinetics. The calculated first-order rate constant (k) at 100 mg L^−1^ was 1.47 × 10^−1^ min^−1^, which was 2.2 times higher than that at 25 mg L^−1^ CoMn_2_O_4_ (6.67 × 10^−2^ min^−1^). The degradation rate of the PBSA was increased together with the amount of the catalyst because the catalytically active sites increased with the amount of CoMn_2_O_4_, and thus more active sites could activate PMS to produce more ROS.

As a precursor to SO_4_^●−^, the concentration of PMS has a great influence on the degradation of pollutants. The effect of different concentrations of PMS on the degradation of the PBSA was also estimated and shown in Figure 4d. The PMS alone could not degrade PBSA when there was no catalyst present. With the increasing amount of PMS, the degradation rate of the PBSA also increased. The k value increased from 0.30 × 10^−1^ to 1.06 × 10^−1^ min^−1^ when the PMS dosage was increased from 125 to 250 mg L^−1^, and this value could be further increased to 1.87 × 10^−1^ min^−1^ with an increased dosage of PMS (500 mg L^−1^). Under certain conditions of the catalyst (50 mg/L) and within a certain range, the PMS amount was increased, leading to the increasing generation of SO_4_^●−^, which consequently accelerated the catalytic degradation rate of the PBSA.

In the CoMn_2_O_4_/PMS system, the removal of the PBSA was also affected by the reaction temperature. As shown in Figure 4e, the higher temperature had a positive effect on the removal of the PBSA. When the dosages of CoMn_2_O_4_ and PMS were individually set as 10 and 250 mg/L, respectively, the degradation rate of the PBSA reached 65.87% after 40 min at 25 °C, and when the temperature was increased to 35 °C, the degradation rate increased to 93.76%. Thereafter, the temperature was further increased to 45 °C, the complete removal of PBSA was observed after 14 min. This indicated that the oxidation reaction was an endothermic reaction. The elevated reaction temperature could significantly increase the degradation rate of the PBSA. The k values at 25, 35, and 45 °C were 2.73 × 10^−^^2^, 4.53 × 10^−^^2^, and 2.30 × 10^−1^ min^−1^, respectively, and thus the activation energy of the reaction was calculated to be 93.12 kJ/mol according to the Arrhenius equation.

The addition of PMS would significantly reduce the solution pH value. The pH of the PBSA solution would reduce from approximately an initial value of 7.3 to 3.4 after the addition of PMS. Thus to examine the effects of real pH on the activation efficiency of CoMn_2_O_4_, the PBSA solution and a certain amount of PMS were added into a conical flask, followed by a pH adjustment with NaOH (0.01 M) or H_2_SO_4_ (0.01 M). As shown in Figure 4f, the PBSA did not degrade well in a more acidic and alkaline environment. The above experimental phenomena could be explained by the following two aspects: on the one hand, the declined performance at an acidic pH may be due to the formation of CoOH^+^, which limits the formation of SO_4_^●−^; whilst the poor performance at an alkaline pH may be associated with the formation of less-reactive Co(OH)_2_ precipitates [40]. On the other hand, the combined effect of the pK_a_ of PMS (9.4, second pK_a_ of its parent acid), the point of zero charge value of CoMn_2_O_4_ (around 7.2), and the pK_a_ of PBSA (pK_a1_ = 4.0 and pK_a2_ = 11.9) might have also contributed to the observed results [46].

**Figure 5:**
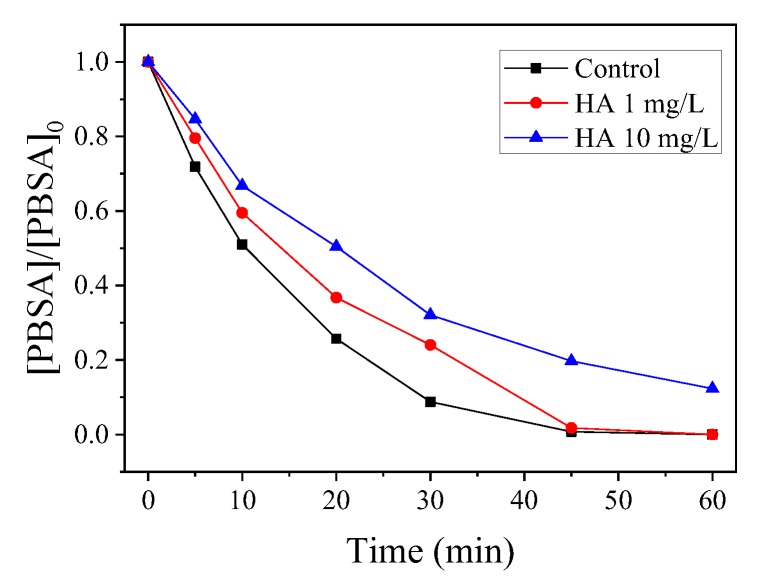
Influence of humic acid (HA) on PBSA removal. Condition: [PBSA] = 5 mg L^−1^; [catalyst] = 50 mg L^−1^; [PMS] = 250 mg L^−1^; *T* = 25 °C; and without pH adjustment.

The dissolved organic matter (DOM) is an important factor because it might quench the free radicals, and thus affect the degradation of the target contaminants. HA represents a typical organic matter in sewage and surface waters. Therefore, the effect of HA on the catalytic PMS oxidation of PBSA by CoMn_2_O_4_ was studied. As shown in Figure 5, the degradation of the PBSA was inhibited when the HA (1–10 mg/L) was added to the system, and this inhibition intensified as the concentration of HA increased. Specifically, 1 mg/L of HA had little effect on the degradation of the PBSA by CoMn_2_O_4_; whereas, when the HA concentration was increased to 10 mg/L, the removal rate of the PBSA in 30 min had significantly decreased from 91.24% to 67.92%. The corresponding k values were 4.80 × 10^−2^ min^−1^ and 3.53 × 10^−2^ min^−1^ at the HA levels of 1.0 and 10.0 mg L^−1^, respectively. The reason may be that HA acts as a degradable organic substance in competition with the PBSA to react with ROS such as free radicals in the system [24].

**Figure 6:**
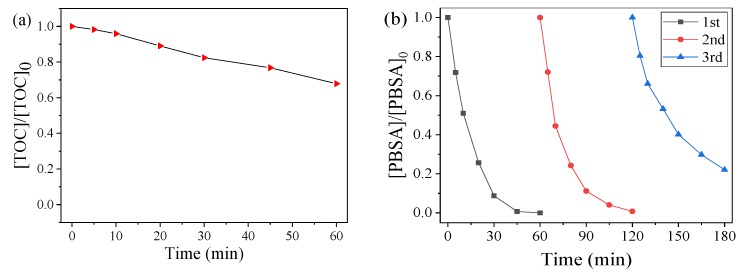
Total organic carbon (TOC) removal of the PBSA oxidized by the CoMn_2_O_4_-activated PMS system (**a**); Degradation of the PBSA using the recycled catalyst (**b**). Conditions: [PBSA] = 5 mg L^−1^; [catalyst] = 50 mg L^−1^; [PMS] = 250 mg L^−1^; *T* = 25 °C; and without pH adjustment.

In this experiment, we measured the TOC removal of the reaction solutions, and the results are shown in Figure 6a. The TOC removal rate of PBSA in the CoMn_2_O_4_/PMS system was 29.59% after 60 min, indicating that the CoMn_2_O_4_/PMS system could not only rapidly degrade the PBSA, but it could also convert it into CO_2_, H_2_O, and other inorganic substances.

In this study, the catalyst was separated and dried after the catalytic degradation reaction. The recyclability of the CoMn_2_O_4_ was evaluated through a multi-cycle experiment of the treated catalyst, and the results are shown in Figure 6b. It could be seen that approximately 80% of the PBSA could be eliminated within 60 min after undergoing the reactions for three repetitions, indicating reasonable stability and reusability of the catalyst.

### 3.3. Activation Mechanism

**Figure 7:**
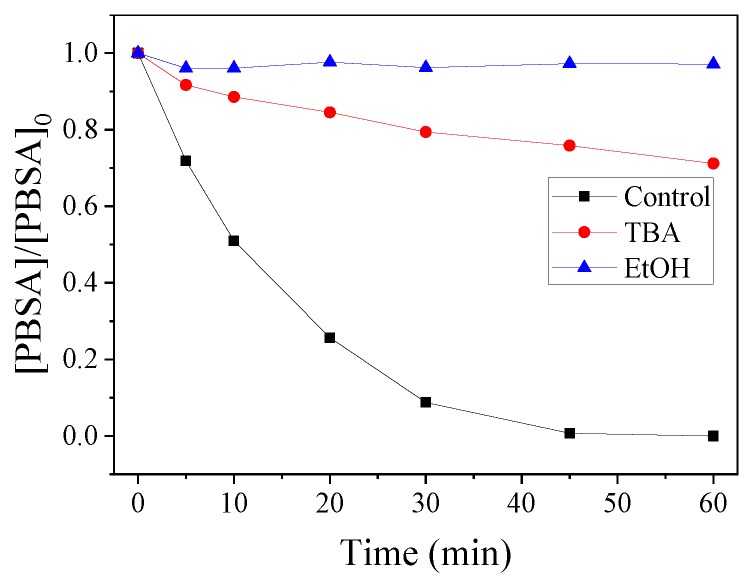
Influences of the different quenchers on PMS oxidation in PBSA degradation. Conditions: [PBSA] = 5 mg L^−1^; [catalyst] = 50 mg L^−1^; [PMS] = 250 mg L^−1^; *T* = 25 °C; [TBA] (or [EtOH])/[PMS] = 1000:1; and without pH adjustment.

**Figure 8:**
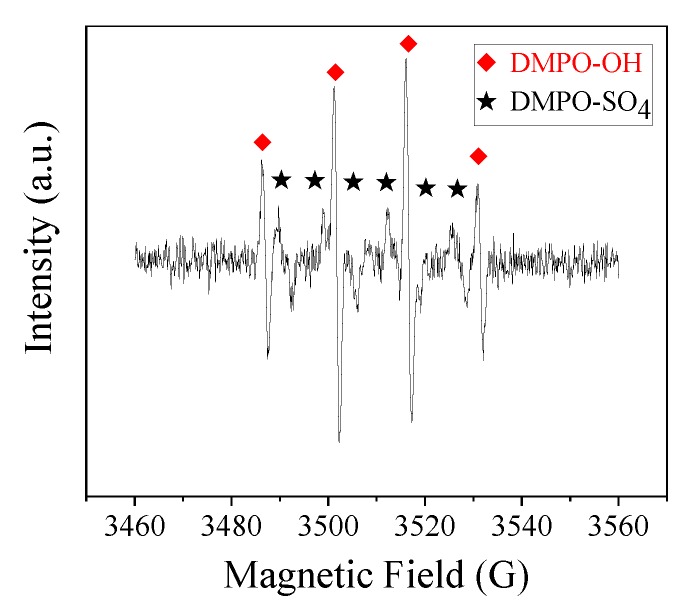
Electron paramagnetic resonance (EPR) spectra of the 5,5-dimethy-1-pyrroline (DMPO) adduct (DMPO–OH and DMPO–SO_4_) formed after five min in the CoMn_2_O_4_/PMS system. Conditions: [catalyst] = 50 mg L^−1^; [PMS] = 250 mg L^−1^; *T* = 25 °C; [DMPO] = 20 mM.

Classical quenching tests were carried out to distinguish the contribution of different ROS in the CoMn_2_O_4_/PMS system. Typically, a certain amount of ethanol (EtOH) or tert-butanol (TBA) was added to the system. As shown in Figure 7, using a 1000:1 molar ratio of EtOH (or TBA) to PMS, the degradation rate was obviously inhibited, indicating the dominated role of the ^●^OH and SO_4_^●−^ in the PBSA removal [47,48]. Moreover, we could judge that the major ROS in the reaction system was mainly ^●^OH, rather than SO_4_^●−^. To further confirm this assumption, EPR tests were carried out, as shown in Figure 8. After five min, DMPO–OH and DMPO–SO_4_ signals were observed, and these results were consistent with the radical quenching tests.

Based on the results from the XPS survey, classical quenching, and EPR tests, a possible mechanism for PMS activation by CoMn_2_O_4_ was proposed under the selected reaction conditions. The specific catalytic reaction process is as follows: PMS first reacts with Co and Mn ions (≡Co^2+^, ≡Mn^2+^, and ≡Mn^3+^) on the surface of CoMn_2_O_4_, producing SO_4_^●−^ (Equations (1)–(3)). Then, it produces SO_4_^●−^ which reacts with water to produce ^●^OH (Equation (4)), and finally, the SO_4_^●−^ and ^●^OH degrade and mineralize the PBSA, as described in Reference [40].

≡Co^2+^ + HSO_5_^−^ → ≡Co^3+^ + SO_4_^●−^ + OH^−^ (1)

≡Mn^2+^ + HSO_5_^−^ → ≡Mn^3+^ + SO_4_^●−^ + OH^−^ (2)

≡Mn^3+^ + HSO_5_^−^ → ≡Mn^4+^ + SO_4_^●−^ + OH^−^ (3)

SO_4_^●−^ + H_2_O → ^●^OH + H^+^ + SO_4_^2−^ (4)

## 4. Conclusions

In this study, a CoMn_2_O_4_ catalyst was synthesized using the sol-gel method, and the obtained catalyst had high catalytic activity for PMS to degrade the PBSA. The degradation of the PBSA was mainly affected by several factors, such as catalyst dosage, PMS concentration, and reaction temperature. In the reaction system, chemical scavenger and electron paramagnetic resonance (EPR) tests proved that ^●^OH and SO_4_^●−^ were the major radicals, playing a dominant role in the PBSA degradation. In addition, the efficient catalytic performance might be attributed to the Co–Mn synergy in the synthesized material. These findings might contribute to the potential application of CoMn_2_O_4_ in SR-AOPs.

## Figures and Tables

**Table 1:** The content of C, O, Co, and Mn on four spots of the CoMn_2_O_4_.

CoMn_2_O_4_	C (wt %)	O (wt %)	Co (wt %)	Mn (wt %)
Spot 1	5.20	38.67	19.05	37.08
Spot 2	4.93	33.19	20.31	41.57
Spot 3	6.25	35.81	19.23	38.70
Spot 4	7.50	32.65	19.34	40.50
Average	5.97	35.08	19.48	39.46

## References

[1] Fisch K., Waniek J.J., Schulz-Bull D.E. (2017). Occurrence of pharmaceuticals and UV-filters in riverine run-offs and waters of the German Baltic Sea. Mar. Pollut. Bull..

[2] He K., Hain E., Timm A., Tarnowski M., Blaney L. (2019). Occurrence of antibiotics, estrogenic hormones, and UV-filters in water, sediment, and oyster tissue from the Chesapeake Bay. Sci. Total Environ..

[3] Grabicova K., Fedorova G., Burkina V., Steinbach C., Schmidt-Posthaus H., Zlabek V., Kroupova H.K., Grabic R., Randak T. (2013). Presence of UV filters in surface water and the effects of phenylbenzimidazole sulfonic acid on rainbow trout (Oncorhynchus mykiss) following a chronic toxicity test. Ecotoxicol. Environ. Saf..

[4] Li A., Law J., Chow C., Huang Y., Li K., Leung K. (2018). Joint effects of multiple UV filters on zebrafish embryo development. Environ. Sci. Technol..

[5] Liu H., Sun P., Liu H., Yang S., Wang L., Wang Z. (2015). Hepatic oxidative stress biomarker responses in freshwater fish Carassius auratus exposed to four commonly benzophenone UV filters. Ecotoxicol. Environ. Saf..

[6] Liu H., Sun P., Liu H., Yang S., Wang L., Wang Z. (2015). Acute toxicity of benzophenone-type UV filters for Photobacterium phosphoreum and Daphnia magna: QSAR analysis, interspecies relationship and integrated assessment. Chemosphere.

[7] Apel C., Joerss H., Ebinghaus R. (2018). Environmental occurrence and hazard of organic UV stabilizers and UV filters in the sediment of European North and Baltic Seas. Chemosphere.

[8] Ao J., Yuan T., Gu J., Ma Y., Shen Z., Tian Y., Shi R., Zhou W., Zhang J. (2018). Organic UV filters in indoor dust and human urine: A study of characteristics, sources, associations and human exposure. Sci. Total Environ..

[9] Sun X., Wei D., Liu W., Geng J., Liu J., Du Y. (2019). Formation of novel disinfection by-products chlorinated benzoquinone, phenyl benzoquinones and polycyclic aromatic hydrocarbons during chlorination treatment on UV filter 2,4-dihydroxybenzophenone in swimming pool water. J. Hazard. Mater..

[10] O’Malley E., O’Brien J.W., Tscharke B., Thomas K.V., Mueller J.F. (2019). Per capita loads of organic UV filters in Australian wastewater influent. Sci. Total Environ..

[11] Liu H., Sun P., He Q., Feng M., Liu H., Yang S., Wang L., Wang Z. (2016). Ozonation of the UV filter benzophenone-4 in aquatic environments: Intermediates and pathways. Chemosphere.

[12] Soto-Vázquez L., Cotto M., Morant C., Duconge J., Márquez F. (2017). Facile synthesis of ZnO nanoparticles and its photocatalytic activity in the degradation of 2-phenylbenzimidazole-5-sulfonic acid. J. Photochem. Photobiol. A.

[13] Du E., Li J., Zhou S., Li M., Liu X., Li H. (2018). Insight into the Degradation of two benzophenone-type UV filters by the UV/H_2_O_2_ advanced oxidation process. Water.

[14] Abdelraheem W.H.M., He X., Komy Z.R., Ismail N.M., Dionysiou D.D. (2016). Revealing the mechanism, pathways and kinetics of UV254nm/H_2_O_2_-based degradation of model active sunscreen ingredient PBSA. Chem. Eng. J..

[15] Abdelraheem W.H.M., He X., Duan X., Dionysiou D.D. (2015). Degradation and mineralization of organic UV absorber compound 2-phenylbenzimidazole-5-sulfonic acid (PBSA) using UV-254 nm/H_2_O_2_. J. Hazard. Mater..

[16] He X., De la Cruz A.A., O’Shea K.E., Dionysiou D.D. (2014). Kinetics and mechanisms of cylindrospermopsin destruction by sulfate radical-based advanced oxidation processes. Water Res..

[17] Liu H., Bruton T.A., Doyle F.M., Sedlak D.L. (2014). In situ chemical oxidation of contaminated groundwater by persulfate: Decomposition by Fe (III)-and Mn (IV)-containing oxides and aquifer materials. Environ. Sci. Technol..

[18] Xiong X., Sun B., Zhang J., Gao N., Shen J., Li J., Guan X. (2014). Activating persulfate by Fe^0^ coupling with weak magnetic field: Performance and mechanism. Water Res..

[19] Ahn Y.Y., Bae H., Kim H.I., Kim S.H., Kim J.H., Lee S.G., Lee J. (2019). Surface-loaded metal nanoparticles for peroxymonosulfate activation: Efficiency and mechanism reconnaissance. Appl. Catal. B Environ..

[20] Zhou Y., Wang X., Zhu C., Dionysiou D.D., Zhao G., Fang G., Zhou D. (2018). New insight into the mechanism of peroxymonosulfate activation by sulfur-containing minerals: Role of sulfur conversion in sulfate radical generation. Water Res..

[21] Liu H., Sun P., Feng M., Liu H., Yang S., Wang L., Wang Z. (2016). Nitrogen and sulfur co-doped CNT-COOH as an efficient metal-free catalyst for the degradation of UV filter BP-4 based on sulfate radicals. Appl. Catal. B Environ..

[22] Sun P., Liu H., Zhai Z., Zhang X., Fang Y., Tan J., Wu J. (2019). Degradation of UV filter BP-1 with nitrogen-doped industrial graphene as a metal-free catalyst of peroxymonosulfate activation. Chem. Eng. J..

[23] Sun P., Liu H., Feng M., Guo L., Zhai Z., Fang Y., Zhang X., Sharma V.K. (2019). Nitrogen-sulfur co-doped industrial graphene as an efficient peroxymonosulfate activator: Singlet oxygen-dominated catalytic degradation of organic contaminants. Appl. Catal. B Environ..

[24] Guerra-Rodríguez S., Rodríguez E., Singh D.N., Rodríguez-Chueca J. (2018). Assessment of sulfate radical-based advanced oxidation processes for water and wastewater treatment: A Review. Water.

[25] Ji Y., Shi Y., Dong W., Wen X., Jiang M., Lu J. (2016). Thermo-activated persulfate oxidation system for tetracycline antibiotics degradation in aqueous solution. Chem. Eng. J..

[26] Ye J., Zhou P., Chen Y., Ou H., Liu J., Li C., Li Q. (2018). Degradation of 1H-benzotriazole using ultraviolet activating persulfate: Mechanisms, products and toxicological analysis. Chem. Eng. J..

[27] Feng M., Cizmas L., Wang Z., Sharma V.K. (2017). Synergistic effect of aqueous removal of fluoroquinolones by a combined use of peroxymonosulfate and ferrate(VI). Chemosphere.

[28] Xu H., Wang D., Ma J., Zhang T., Lu X., Chen Z. (2018). A superior active and stable spinel sulfide for catalytic peroxymonosulfate oxidation of bisphenol S. Appl. Catal. B Environ..

[29] Alexopoulou C., Petala A., Frontistis Z., Drivas C., Kennou S., Kondarides D.I., Mantzavinos D. (2019). Copper phosphide and persulfate salt: A novel catalytic system for the degradation of aqueous phase micro-contaminants. Appl. Catal. B Environ..

[30] Ding Y., Zhu L., Wang N., Tang H. (2013). Sulfate radicals induced degradation of tetrabromobisphenol A with nanoscaled magnetic CuFe_2_O_4_ as a heterogeneous catalyst of peroxymonosulfate. Appl. Catal. B Environ..

[31] Deng J., Ya C., Ge Y., Cheng Y., Chen Y., Xu M., Wang H. (2018). Activation of peroxymonosulfate by metal (Fe, Mn, Cu and Ni) doping ordered mesoporous Co_3_O_4_ for the degradation of enrofloxacin. RSC Adv..

[32] Wang Y., Sun H., Ang H., Tadé M.O., Wang S. (2014). Magnetic Fe_3_O_4_/carbon sphere/cobalt composites for catalytic oxidation of phenol solutions with sulfate radicals. Chem. Eng. J..

[33] Li R., Cai M., Xie Z., Zhang Q., Zeng Y., Liu H., Liu G., Lv W. (2019). Construction of heterostructured CuFe_2_O_4_/g-C_3_N_4_ nanocomposite as an efficient visible light photocatalyst with peroxydisulfate for the organic oxidation. Appl. Catal. B Environ..

[34] Duan X., Su C., Miao J., Zhong Y., Shao Z., Wang S., Sun H. (2018). Insights into perovskite-catalyzed peroxymonosulfate activation: Maneuverable cobalt sites for promoted evolution of sulfate radicals. Appl. Catal. B Environ..

[35] Li J., Hussain A., Li D., Yang M., Xu S. (2017). Catalytic performance of graphene-bimetallic composite for heterogeneous oxidation of acid orange 7 from aqueous solution. Environ. Sci. Pollut. Res..

[36] Shi P., Su R., Wan F., Zhu M., Li D., Xu S. (2012). Co_3_O_4_ nanocrystals on graphene oxide as a synergistic catalyst for degradation of Orange II in water by advanced oxidation technology based on sulfate radicals. Appl. Catal. B.

[37] Feng M., Qu R., Zhang X., Sun P., Sui Y., Wang L., Wang Z. (2015). Degradation of flumequine in aqueous solution by persulfate activated with common methods and polyhydroquinone-coated magnetite/multi-walled carbon nanotubes catalysts. Water Res..

[38] Zhang X., Feng M., Qu R., Liu H., Wang L., Wang Z. (2016). Catalytic degradation of diethyl phthalate in aqueous solution by persulfate activated with nano-scaled magnetic CuFe_2_O_4_/MWCNTs. Chem. Eng. J..

[39] Yao Y., Cai Y., Wu G., Wei F., Li X., Chen H., Wang S. (2015). Sulfate radicals induced from peroxymonosulfate by cobalt manganese oxides (CoxMn_3_−xO_4_) for Fenton-like reaction in water. J. Hazard. Mater..

[40] Li C., Chen C., Lu J., Cui S., Li J., Liu H., Li W., Zhang F. (2018). Metal organic framework-derived CoMn_2_O_4_ catalyst for heterogeneous activation of peroxymonosulfate and sulfanilamide degradation. Chem. Eng. J..

[41] Al-Anazi A., Abdelraheem W.H., Han C., Nadagouda M.N., Sygellou L., Arfanis M.K., Falaras P., Sharma V.K., Dionysiou D.D. (2018). Cobalt ferrite nanoparticles with controlled composition-peroxymonosulfate mediated degradation of 2-phenylbenzimidazole-5-sulfonic acid. Appl. Catal. B Environ..

[42] Pang X., Guo Y., Zhang Y., Xu B., Qi F. (2016). LaCoO_3_ perovskite oxide activation of peroxymonosulfate for aqueous 2-phenyl-5-sulfobenzimidazole degradation: Effect of synthetic method and the reaction mechanism. Chem. Eng. J..

[43] Rossetti L., Bonelli B., Ramis G., Bahadori E., Nasi R., Aronne A., Esposito S. (2018). New insights into the role of the synthesis procedure on the performance of co-based catalysts for ethanol steam reforming. Top. Catal..

[44] Esposito S. (2019). “Traditional” sol-gel chemistry as a powerful tool for the preparation of supported metal and metal oxide catalysts. Materials.

[45] Lavela P., Tirado J.L., Vidal-Abarca C. (2007). Sol–gel preparation of cobalt manganese mixed oxides for their use as electrode materials in lithium cells. Electrochim. Acta.

[46] Zhang X., Feng M., Wang L., Qu R., Wang Z. (2017). Catalytic degradation of 2-phenylbenzimidazole-5-sulfonic acid by peroxymonosulfate activated with nitrogen and sulfur co-doped CNT-COOH loaded CuFe_2_O_4_. Chem. Eng. J..

[47] Yang Y., Pignatello J.J., Ma J., Mitch W.A. (2014). Comparison of halide impacts on the efficiency of contaminant degradation by sulfate and hydroxyl radical-based advanced oxidation processes (AOPs). Environ. Sci. Technol..

[48] Guan Y., Ma J., Ren Y., Liu Y., Xiao J., Lin L., Zhang C. (2013). Efficient degradation of atrazine by magnetic porous copper ferrite catalyzed peroxymonosulfate oxidation via the formation of hydroxyl and sulfate radicals. Water Res..

